# Evaluation of bone regenerative and bioactive potential of β-TCP/PLLA/PGA nanobiomaterial in conjunction with concentrated conditioned media from rapidly expanding clone (REC) of mesenchymal stem cells: A pilot animal study

**DOI:** 10.1371/journal.pone.0332531

**Published:** 2025-10-09

**Authors:** Ankhtsetseg Shijirbold, Mrunalini Ramanathan, Masako Fujioka-Kobayashi, Nithish Sankepally, Yuhei Matsuda, Yumi Matsuzaki, Yuki Fujita, Takahiro Kanno

**Affiliations:** 1 Department of Oral and Maxillofacial Surgery, Shimane University Faculty of Medicine, Izumo, Shimane, Japan; 2 Department of Life Science, Shimane University Faculty of Medicine, Izumo, Shimane, Japan; 3 Department of Developmental Biology, Shimane University Faculty of Medicine, Izumo, Shimane, Japan; Changchun Institute of Applied Chemistry, Chinese Academy of Sciences, CHINA

## Abstract

Maxillofacial bone defects present substantial challenges in reconstruction because of their impact on function and aesthetics. Mesenchymal stem cells (MSCs) have been used for therapeutic purposes; however, various technical issues limit their application. A variant of MSCs called “rapidly expanding clone” (REC) was introduced using the superselection method. Conditioned media (CM) from stem cells have shown promising regenerative efficacy and can prevent host immunogenic reactions. We hypothesized that the bone regeneration potential of β-TCP/PLLA/PGA nanobiomaterial scaffold combined with concentrated CM-derived REC MSCs could enhance the regeneration process. For comparative evaluation, plain CM, 1 time concentrated REC-CM (1 × REC-CM), and 10 times concentrated REC-CM (10 × REC-CM) subgroups were generated. The quantity of growth factors in each CM group was assessed using enzyme-linked immunosorbent assay. β-TCP/PLLA/PGA scaffold implantation with REC-CM was performed in rats with critical-sized mandibular defects. Newly regenerated bone was evaluated using the bone volume-to-total volume (BV/TV) ratio, bone mineral density (BMD), and defect margin regrowth, and bioactivity was checked for Runx2, LepR, osteocalcin, periostin accumulation, and deposition at weeks 1 and 4. 10 × REC-CM promoted angiogenesis by significantly elevating TGF-β1 (p = 0.0002) and VEGF (p = 0.007) levels, facilitating vascularization and matrix deposition. The 10 × REC-CM group demonstrated substantially higher BV/TV at week 4 and new bone regeneration in the inner defect region at both time points (p = 0.002; p = 0.04) than the other groups. Significant elevation in the levels of osteogenic markers, such as Runx2 (p = 0.001; p = 0.05), LepR (p = 0.002; p = 0.001), osteocalcin (p = 0.001), and periostin, was observed in the 10 × REC-CM group, indicating good osteoinductivity. These findings highlight the synergistic effect of β-TCP/PLLA/PGA scaffold and REC-CM in promoting effective bone regeneration. The therapeutic potential of REC-CM-enhanced scaffolds in maxillofacial and bone reconstruction is supported by the findings presented in this study. This study demonstrates the upregulation and early phase bone regeneration upon application of 10x REC-CM with β-TCP/PLLA/PGA nanobiomaterial.

## Introduction

Maxillofacial defects are characterized by iatrogenic loss or abnormal bone development within the facial and jaw regions and can be broadly classified into congenital and acquired categories [1]. These defects can significantly impair an individual’s functional and aesthetic aspects, often resulting in compromised masticatory function, speech, and overall facial appearance [1]. The absence of adequate bone structure can impair vital functions, such as mastication, respiration, and phonation, while also contributing to psychological distress due to changes in facial aesthetics [2]. Such defects continue to pose substantial challenges for reconstruction in maxillofacial surgery, and their restoration is essential for the recovery of function and appearance [3]. Acquired bone defects are traditionally managed using autografts, allografts, and xenografts to stimulate bone regeneration [4]. Autografts, often referred to as the ‘gold standard” owing to their osteoconductive and osteoinductive properties, provide an ideal environment for new bone formation [5]. However, despite these advantages, autografts present notable drawbacks, primarily their invasiveness, which can lead to donor-site morbidity and additional patient discomfort. Allografts derived from donor bone tissue eliminate the need for a secondary surgical site; however, they are prone to immunogenic responses and risk of disease transmission [6]. Additionally, studies have documented high failure rates of allografts in long-term use as they undergo resorption and show limited integration within the host tissue [7]. Xenografts sourced from non-human species and alloplastic materials are considered alternatives, although they lack regenerative potential and integration properties.

Because of these limitations, bone-like structured materials have been used in tissue engineering [8,9]. Synthetic bone substitutes that have shown potential are hydroxyapatite (HA) and β-Tricalcium Phosphate (β-TCP), a scaffold composed of purified inorganic tricalcium phosphate. β-TCP exhibits osteoinductive and osteoconductive supports, which support cellular growth and bone regeneration [10]. It has an interconnected porous structure that facilitates fibrovascularization, mimics the natural osteogenic properties of bone, and provides a supportive framework for new tissue formation [11]. However, despite its advantages, β-TCP has inherent brittleness. Owing to its lack of pliability, β-TCP is applicable only to small bone defects in the maxillofacial region, where precision shaping is less critical. While β-TCP can promote bone growth effectively in smaller applications, its structural limitations hinder its suitability for larger or load-bearing defects [12].

Bioresorbable unsaturated polyester materials have evolved significantly and are classified into four main generations based on their composition, chronology, and physicochemical and biological properties [13]. Second-generation materials have emerged using combinations of polyglycolic acid (PGA), PLLA (l-lactic acid (PLLA), and poly-d-lactic acid (PDLA). Modulation of the strength and resorption rate to align with the tissue growth requirements is possible with PLLA/PGA composites. Despite these advances, PLLA/PGA biomaterials lack osteoinduction, which is critical for effective bone healing [14]. Although PLLA provides adequate strength for some applications, neither PLLA nor PGA alone is suitable for load-bearing regions, necessitating reinforcement with additional agents to improve their mechanical properties.

William Gilbert first proposed the idea of electrospinning in 1600’s literature [15]. It was not until the early 1990s that Reneker and Rutledge reinvented the electrospinning technique to produce nanomaterials from polymers down to the 3–5000 nanometer scale [15,16]. This method is particularly effective for generating porous scaffolds with high surface area-to-volume ratios, which are ideal for promoting cell adhesion, proliferation, and differentiation [17,18]. To obtain flexibility and easy handling, β-TCP was combined with PLLA/PGA to form β-TCP/PLLA/PGA nanobiomaterial through an electrospinning technique. The properties and bone formation mechanism emphasizing osteoinduction and osteoconduction of β-TCP/PLLA/PGA nanobiomaterial have been elucidated in a previous publication [12].

Mesenchymal stem cells (MSCs) have been used alongside scaffolds in experimental studies and have shown promising results in the regeneration of various tissues, such as cartilage, bone, and nerves [19]. While MSC-mediated regeneration has shown good experimental outcomes, its application in mainstream therapeutic settings is limited owing to limitations in extraction methods or contamination, leading to decreased quality and impurity, which pose substantial concerns for mass production [20,21]. A highly purified variant of MSCs called “rapidly expanding clone” (REC), derived from human bone marrow mononuclear cell, identified by its potential to organize into colony-forming unit fibroblasts, was isolated using LNGFR+THY-1+ and VCAM-1 expression by researchers from Shimane University [22]. Color staining of bone marrow extract cells revealed that human-MSCs exist only in the LNGFR+THY-1 + cell subpopulation, with the highest number of colony-forming unit fibroblasts [22]. Notably, REC cells reach confluence within 2 days of *in vitro* culture and can undergo passage several times. REC cells are commercially available for therapeutic purposes under the brand name PuREC. Very few studies employing REC cells for tissue regeneration in animal models have demonstrated favorable outcomes [23,24].

The viability of implanted stem cells inside the host tissue is a significant concern, as it has been reported that cells are gradually depleted from the injection site within 2–4 weeks [25]. Human stem cells can produce immunogenic reactions in animals, leading to cell death; therefore, daily anti-immune injections are necessary [26]. Stem cells possess paracrine activity and secrete growth factors and proteins *in vitro*, including secretome vesicles, into the media in which they grow. The media collected a few days after the culture is called “conditioned media” (CM), which can be either freeze-stored or used afresh to promote beneficial effects on bone growth. Lyophilized CM from the dental pulp has been used to induce bone formation in critically sized calvarial defects in rats [19]. Concentrated CM has shown promising efficacy in clinical settings, particularly with regard to skin regeneration. To prevent the disadvantages associated with the direct injection of REC cells, mainly for the prevention of host autoimmune reactions, we decided to employ a novel approach by testing the efficacy of 1 time (1×) and 10 times (10×) concentrated REC-CM in promoting new bone regeneration. To the best of our knowledge, this is the first study to use REC-CM as a scaffold *in vivo* to assess new bone regeneration and bioactivity induction.

We hypothesized that β-TCP/PLLA/PGA nanobiomaterial, in conjunction with 1× and 10 × concentrated REC-CM, possesses good bone regeneration potential through the combined effects of growth factor supplementation as well as the attraction of osteogenic cells through osteoinduction and bone deposition by osteoconduction. We designed an *in vivo* animal study to analyze the results of a critically sized bone defect in the rat mandible.

## Materials and methods

### β-TCP/PLLA/PGA nanobiomaterial

**Fig 1:**
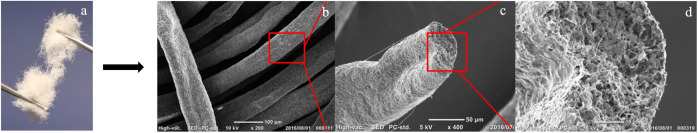
Macroscopic and microscopic images of β-TCP/PLLA/PGA. (a) Image depicts the macroscopic appearance and structure of the β-TCP/PLLA/PGA electrospun nanobiomaterial. Images (b), (c), and (d) denote the SEM images of β-TCP/PLLA/PGA electrospun cotton-like structured nanobiomaterial in ×200, × 400, and ×2000 magnifications, respectively.

β-TCP/PLLA/PGA, or ReBOSSIS-MT (ORTHOReBIRTH, Kanagawa, Japan), commercially available an electrospun cotton-like bioresorbable material composed of 70% βTCP and 30% PLLA/PGA by weight, was used in this study. β-TCP particles with a size of 1–5 μm were interspersed in the PLLA/PGA fibers. The electrospun material was fabricated using the following steps. A specific amount of the materials mentioned above was melted, and meticulous kneading was performed to create a homogenous mixture. Chloroform was then added to the mixture, and subsequent electrospinning at 25 kV was performed to generate cotton-like fibers. The volume of β-TCP/PLLA/PGA nanobiomaterial used in this study was 5 mg. Each 5 mg of material was precisely measured, sterilized, and packed in separate containers by ORTHOReBIRTH. Scanning electron microscopy (SEM) images of β-TCP/PLLA/PGA nanobiomaterial are shown in Fig 1.

### Preparation of REC-concentrated CM

REC cells were obtained from PuREC® Corporation (Department of Life Sciences, Shimane University Faculty of Medicine) and is commercially available. Rapidly expanding clone of mesenchymal stem cells (MSC’s) were derived from human (cadaver) bone marrow mononuclear cells. Bone marrow cells were collected traditionally by flushing the bone marrow or collagenase digestion of crushed bone [22]. The REC cells were isolated using LNGFR+THY–1 + VCAM–1hi+ and cultured in tissue culture plates containing low-glucose Dulbecco’s modified Eagle’s medium (Wako Pure Chemical Corp., Osaka, Japan) supplemented with 20% fetal bovine serum (Hyclone, South Logan, UT, USA), 10 mM 4-(2-hydroxyethyl)-1-piperazineethanesulfonic acid (HEPES; Nacalai, Kyoto, Japan), and 1% penicillin/streptomycin (Wako Pure Chemical Corp., Osaka, Japan). REC were cultured for 2 days to allow the release of growth factors. The REC cells were passaged on day 3 after reaching 90% confluence. The CM from the REC was then collected carefully and stored in a deep freezer at a temperature of −80°C. During the experiment, REC-CM was thawed and concentrated 10 times using a centrifugal concentrator with Ultracel^®^ membrane with 3 kDa MWCO (Amicon^®^ Ultra, Merck KGaA, Darmstadt, Germany). The volume of REC-CM (10 mL) was reduced to 1 mL. Prior to implantation, the β-TCP/PLLA/PGA scaffold was imbued with REC-CM.

### Enzyme-linked immunosorbent assay (ELISA) study

ELISA was employed to evaluate the levels of transforming growth factor-beta 1 (TGF-β1) and vascular endothelial growth factor (VEGF) in 1× and 10 × REC-CM. For this assay, Human VEGF Quantikine® ELISA Kit (DY293B-05; R&D Systems Inc., USA) and Human TGF-β1 Quantikine® ELISA Kit (SB100C; R&D Systems Inc., USA) were used. These kits were specifically designed to quantitatively determine their targets in biological samples with high sensitivity and reproducibility. All experiments were conducted in strict accordance with the manufacturer’s protocol.

The absorbance of each well was measured at 450 nm, the primary detection wavelength, and 540 nm and used for background subtraction using a SoftMax Pro 6.4 microplate reader (Molecular Devices, LLC, USA). Wavelength correction was performed to account for optical imperfections in the microplate, ensuring a higher accuracy in the quantification process.

The concentration of TGF-β1 and VEGF in each sample was calculated by comparing the corrected absorbance values to the standard curve generated for each analyte. A standard curve was constructed by plotting the standard optical density (OD) against known concentrations, and linear regression or four-parameter logistic curve fitting was applied depending on the kit’s recommendations. To ensure precision and reliability, all measurements were performed in duplicate or triplicate, and control samples were included in each experiment. Data were analyzed and interpreted according to the manufacturer’s guidelines provided with the kits.

### Animal experimental sequence

**Fig 2:**
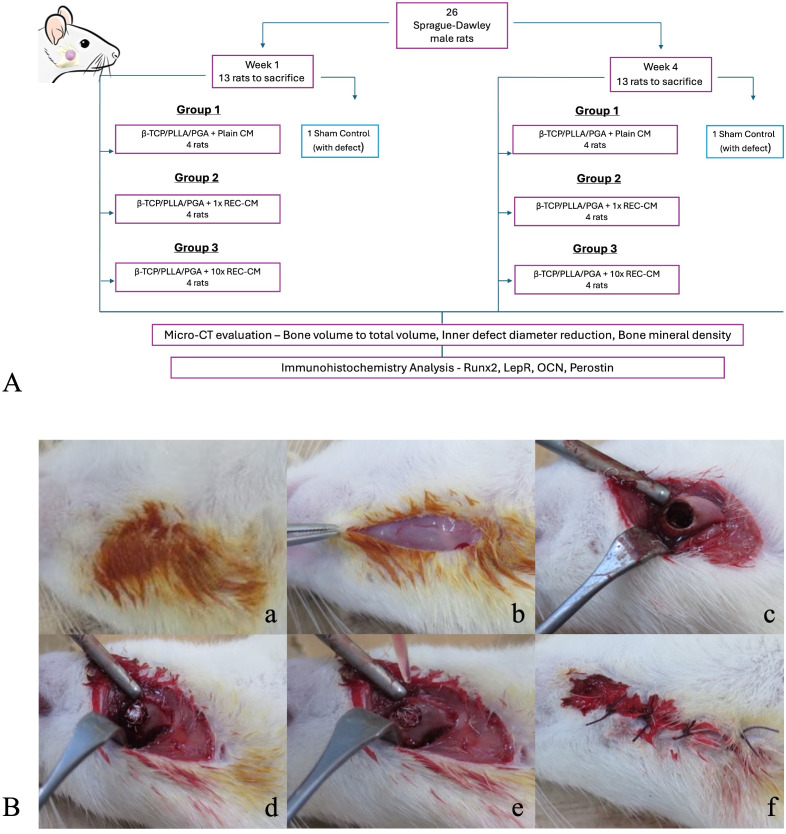
Experimental workflow and surgical procedure. (A) Detailed flow chart of the experiment. (B) From left to right: (a) Disinfection of the surgical area on the submandibular region; (b) Skin incision exposing muscular layer; (c) 4 mm defect creation above the mandibular angle; (d) 5g of β-TCP/PLLA/PGA into the defect; (e) REC-CM was injected via pipette into the scaffold; (f) Wound closure.

Twenty-six Sprague-Dawley (SD) male Sprague-Dawley rats with an average weight of 355–365 g were obtained for our animal study, with the approval of the Institutional Review Board (IZ4-33-3). The experiments were performed according to an established protocol for the care and use of laboratory animals and were approved by the Institutional Animal Care and Use Committee (IACUC) of Shimane University (12). The rats were divided into three subgroups: four rats in the β-TCP/PLLA/PGA–plain CM group, four rats in the β-TCP/PLLA/PGA–1 × REC-CM, four rats in the β-TCP/PLLA/PGA–10 × REC-CM media group, and two sham controls with defects. Fig 2A illustrates the experimental methodology. The surgical procedures were performed under aseptic conditions. SD rats were anesthetized with a 3-mixture anesthetic solution that consisted of medetomidine hydrochloride (0.15 mg/kg), midazolam (2 mg/kg), and butorphanol (2.5 mg/kg) with appropriately diluted sterile water. The anesthetic solution was injected intraperitoneally to minimize pain and distress. The external skin on the right side of the mandible was disinfected with povidone-iodine. A 1-cm long longitudinal incision was made in the submandibular region, exposing the buccal surface of the mandibular bone through careful dissection of the soft tissue layers and muscles. Using a trephine bur, we created a 4 mm diameter bi-cortical, non-self-repairing critical-size defect above the mandibular angle region. For the purpose of standardization, the critical sized defect was created only on the right side in our research. Each defect was filled with 5 mg TCP/PLLA/PGA. The rats in the study group received either Plain CM or 1× or 10 × REC-CM and β-TCP/PLLA/PGA on the right side of the mandible. Precisely 10μl of Plain CM, 1x, and 10x REC-CM was injected into the filled β-TCP/PLLA/PGA scaffold using a pipette Fig 2B. Antibiotics and analgesic injections were injected after surgery. About one–two hours after the procedure, the rats emerged from the anesthesia, resumed their regular activities, and showed signs of a normal appetite. Animals were closely monitored for signs of pain, distress, or health complications. The weight and overall health of the rats were routinely checked. All the rats were alive and healthy until the day of sacrifice. At the sacrifice time euthanasia was performed through perfusion using formalin and saline at two time points: weeks 1 and week 4, using approved humane methods, followed by tissue harvesting for analysis. The IACUC confirmed that all procedures complied with ethical and welfare standards, and all efforts were made to minimize the number of animals used and their suffering. The right hemi-mandible containing β-TCP/PLLA/PGA was removed upon sacrifice and placed in containers filled with 10% neutral-buffered formalin solution. Each container was labeled and packed for Micro-Computed Tomography (Micro-CT) and immunohistochemistry (IHC) analyses. All four specimens per group at each time point were used to assess parameters. The Sham group was used to demonstrate that the critical-size defect could not heal without further intervention and hence was excluded from the micro-CT and IHC analyses.

### Micro-CT: procedure and analyses

A CosmoScan FX micro-CT scanner (Rigaku Corporation, Tokyo, Japan) was used to scan the mandibles of the harvested rats. Scanning was spent scanning. 90 kV of voltage and a current of 88 A were used. The resolution was 20 m, with a matrix of 512 × 512 × 512 and a field of view of 10.24 × 10.24 × 10.24 mm. The specimens were scanned using calcium hydroxyapatite (CaHA) phantoms (QRM, Moehrendorf, Germany) containing five cylinders at densities of 0, 50, 200, 800, and 1200 g/cm3. In-Vivo Science Inc. (Kanagawa, Japan) performed the micro-CT scanning.

Bone mineral density (BMD), defined as the volumetric density of calcium hydroxyapatite (CaHA) in biological tissue (g/cm^3^), was estimated for newly generated bone using CTAn version 1.19+ (Skyscan, Bruker, Kontich, Belgium). Calibration was performed against the attenuation coefficient (AC, mm ⁻ ¹), a direct measure of X-ray absorption, as X-ray absorption in mineralized tissues is primarily influenced by CaHA. Two phantoms (800 and 1200 g/cm^3^ cylinders) were used for AC calibration, and the relationship between AC and mass density was defined by the formula: BMD (g/cm^3^) = AC–0.482/0.2275. The Digital Imaging and Communications in Medicine (DICOM) micro-CT data of the specimens were processed using CTAn software with regions of interest (ROI) encompassing 50–70 slices and adjusted to defect size. The ROI images were saved in separate folders. Standardized circular ROIs 4 mm in diameter were consistently applied to all samples within the critical defect regions, and the BMD values were calculated.

The bone volume-to-total volume (BV/TV) ratio and inner defect diameter reduction were analyzed using Fiji software (ImageJ; NIH, Bethesda, MD, USA) with TransformJ and BoneJ plugins. TransformJ was used to accurately angulate the CT slides, enabling visualization of the defect region as a spheroid. For the BV/TV calculation, DICOM slides containing nanobiomaterials from the anterior to posterior sections were selected. A 4 mm circular region of interest (ROI) simulating the defect area was outlined using an elliptical tool, and the chosen slides were duplicated into a new stack. The ROI images were then binarized and BoneJ was used to calculate the area/volume fraction, generating results in the log window (S1 Fig). The middle slide from the micro-CT dataset was used to assess the inner defect diameter. A 4-mm circular defect was recreated, and a polygonal selection tool was used to trace and measure the new bone within the defect area using the ROI Manager. The BV/TV ratio and inner defect measurements were focused on critical defect regions of the mandible, ensuring a standardized evaluation.

### Histology, IHC staining, and evaluation

Hematoxylin and eosin (H&E) staining was performed for all specimens, including those from the sham control group, whereas IHC staining was conducted only for the study group specimens. After micro-CT imaging, samples were fixed in 10% formalin and sent to Sept. Sapie Co., Ltd. (Tokyo, Japan) for staining. Mandibular samples were sectioned into 5 μm thick slices, with two sections stained using H&E as reference slides and another three sections subjected to IHC staining targeted specific markers to evaluate runt related transcription factor x (Runx2), osteocalcin (OCN), leptin receptor (LepR), and periostin. Deparaffinization was performed using xylene, followed by rehydration using an ethanol gradient, and washing with PBS. Antigen retrieval involved heat treatment with sodium citrate buffer at 80–90°C for 8 minutes. Nonspecific binding was blocked using 5% bovine serum albumin (BSA) in PBS and endogenous peroxidase activity was suppressed using 3% hydrogen peroxide. Primary antibody incubation was performed at room temperature for 50 min using the following specific antibodies: anti-Runx2 rabbit polyclonal antibody (1:300; Abcam: ab23981), anti-human osteocalcin monoclonal antibody clone (1:3000; BIO-RAD 0400−0041), anti-LepR rabbit polyclonal antibody (1:150; Proteintech, 20966–1-AP), and anti-periostin rabbit polyclonal antibody (1:800; Abcam: ab14041). Sections were washed with PBS and incubated with Histofine Simple Stain Rat MAX-PO (MULTI) (Nichirei Biosciences Inc., Tokyo, Japan; 414191) secondary antibodies for 30 min. A diaminobenzidine (DAB) substrate was used to visualize antigen-antibody binding, resulting in a brown precipitate, which was then counterstained with Meyer’s hematoxylin, dehydrated, and mounted for analysis. Negative controls were prepared by omitting the primary antibody to confirm staining specificity.

### IHC-OD analysis

All specimens were evaluated using a BX43 light microscope (Olympus Corporation, Tokyo, Japan) equipped with an Olympus D21-CB digital photo system. ImageJ software, equipped with the IHC Profiler plugin, was used to assess the OD of IHC staining for Runx2, OCN, LepR, and periostin. Three specimens from each group were analyzed at each time point, with three images captured per specimen at 20x magnification. The IHC Profiler plugin uses the DAB color deconvolution algorithm to generate semi-quantitative OD scores that reflect the intensity of antigen expression. This method ensured a detailed and reproducible assessment of IHC staining across all samples (S2 Fig). These scores were converted into quantitative values of positive cells using the following standardized formula:

$\begin{array}{cc} \text{IHC OD score}= & \text{Percentage contribution of high positive}\\ & \;\;\text{+ Percentage contribution of positive ×\textasciitilde{}2}\\ & \;\;\;\;\text{ + Percentage contribution of low positive × 1} \end{array}$

### Statistical analysis

Statistical analysis was performed using SPSS software version 27.0 for MAC OS (IBM Corp., Armonk, NY, USA). GraphPad Prism 10 software version 10.5.0 (673) for MAC OS (GraphPad Software LLC, Boston, MA, USA) was used to generate the graphs demonstrated in the following results section. Shapiro-Wilk testing was performed on all datasets to identify normality (S1 and S2 Tables). The results of BMD, BV/TV, and inner defect reduction, as well as IHC staining for Runx2, OCN, LepR, and periostin between the plain media and 1× and 10 × concentrated REC-CM groups at weeks 1 and 4 were analyzed using one-way ANOVA, followed by a post hoc Bonferroni test to reduce error probability. Student’s t-test for independent samples was performed in the comparison of concentration in TGF- β1 and VEGF. p < 0.05 indicated statistical significance.

## Results

### ELISA result

#### TGF-β1 concentration.

**Fig 3:**
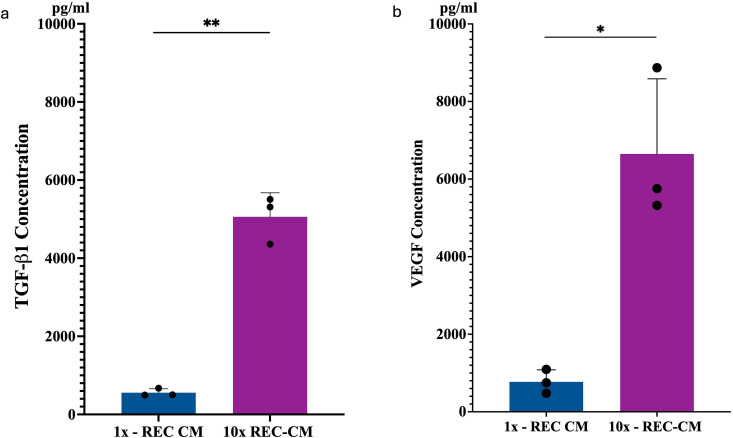
ELISA result graph. **(a)** TGF-β1 concentration level in 100µl between 1 × REC-CM and 10 × REC-CM, with significant differences shown on the graph. (**b)** VEGF concentration level in 200µl between 1 × REC-CM and 10 × REC-CM (**p < 0.0002; *p < 0.05).

The ELISA results for TGF-β1 (transforming growth factor beta 1), a critical cytokine in bone remodeling and regeneration, reveal a significant difference between the 1 × REC-CM and 10 × REC-CM groups. The 1 × REC-CM group exhibited low TGF-β1 concentrations, reflecting a limited potential to stimulate osteogenesis. In contrast, the 10 × REC-CM group showed a markedly higher concentration of TGF-β1, with a statistically significant difference (p < 0.0002) compared to the 1 × REC-CM group. The elevated TGF-β1 levels in the 10 × REC-CM group suggest enhanced osteogenic activity and more substantial support for extracellular matrix production, osteoblast differentiation, and bone regeneration (Fig 3a).

#### VEGF concentration.

The ELISA results for VEGF (vascular endothelial growth factor), a key angiogenic factor involved in bone regeneration, also show a significant dose-dependent difference between the groups. The 1 × REC-CM group showed low VEGF levels, indicating limited angiogenic stimulation. However, the 10 × REC-CM group demonstrated significantly higher VEGF concentrations than a significant difference (p < 0.007) compared to the 1 × REC-CM group. Elevated VEGF levels in the 10 × REC-CM group highlighted its capacity to promote vascularization, a critical process for supporting nutrient delivery, scaffold integration, and enhanced bone regeneration (Fig 3b).

### Assessment of newly regenerated bone

**Fig 4:**
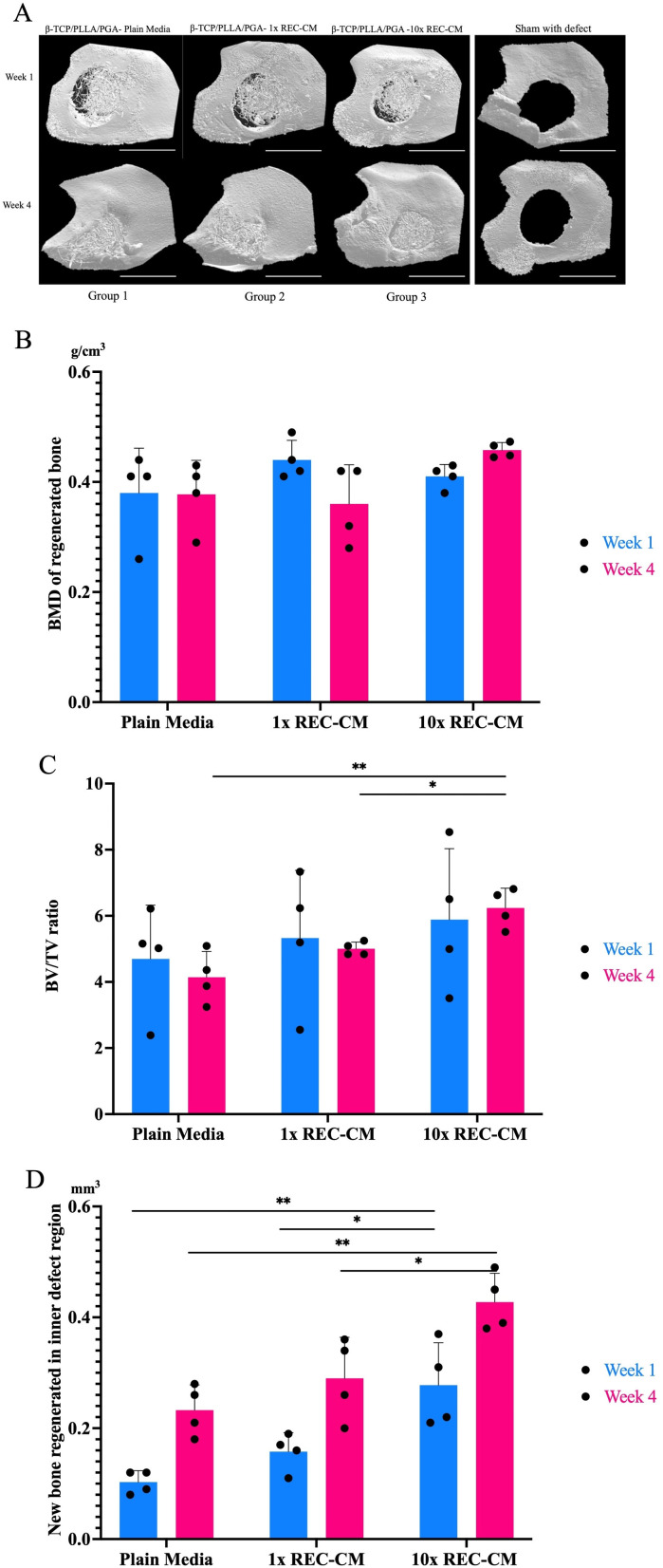
Micro-CT imaging and quantitative analysis of BMD, BV/TV, and new bone regeneration in the inner defect region from harvested rat mandible. (A): From left to right: Micro-CT image comparison between Group 1, Group 2, and Group 3 in 2 time points. Steady rim closure can be seen by Week 4; Micro-CT image of Sham with defect shows insignificant bone regeneration. For all groups, the standard defect size was 4 mm. Volume of the biomaterial applied: β-TCP/PLLA/PGA - 5 mg, and CM – 10μl across all groups. Scale bar = 1 mm. (B): The difference in the BMD of regenerated trabecular bone at two set time points between the 3 groups. The graph shows sig. difference in β-TCP/PLLA/PGA–10 × REC-CM at week 4. (C): The graph shows the BV/TV ratio between the β-TCP/PLLA/PGA–plain media, β-TCP/PLLA/PGA–1 × REC-CM, and β-TCP/PLLA/PGA–10 × REC-CM groups at two time points. The difference was evident at week 4. (D): New bone regeneration in the inner defect region of β-TCP/PLLA/PGA–10 × REC-CM had more defect rim closer in both time points than the two other groups (**p < 0.0002; *p < 0.05).

Micro-CT data from rat mandibular specimens at weeks 1 and 4 were used to evaluate the new bone regeneration in relation to the scaffold with both concentrations (Fig 4A). The new bone was evident macroscopically on the exterior, between the β-TCP/PLLA/PGA fibers. In the β-TCP/PLLA/PGA–10 × REC-CM group), solid bone formation from the defect’s rim was noticeable, and the volume of new bone grew steadily. There was little change in the Sham group between Weeks 1 and 4. But in Week 4, a tiny bony portion is evident.

### BMD

At both time points, the BMD showed no significant differences among the three groups. At week 4, we observed a tendency for comparable bone formation albeit a slightly higher new bone regeneration in the 10x-REC-CM group (Fig 4B).

### BV/TV result

At week 1, all three groups exhibited relatively comparable BV/TV ratio, with no statistically significant difference among the groups. The BV/TV ratio significantly differed among the groups at week 4. The β-TCP/PLLA/PGA–plain media group showed slightly increased bone formation compared to week 1 but remained the lowest among all groups. The β-TCP/PLLA/PGA–10 × REC-CM group demonstrated the highest bone regeneration, with the significance of the β-TCP/PLLA/PGA–plain media group (p = 0.002) and β-TCP/PLLA/PGA–1 × REC-CM group (p = 0.04) (Fig 4C).

### Inner defect diameter reduction

At week 1, the β-TCP/PLLA/PGA–10 × REC-CM group exhibited the most significant reduction in the 4-mm defect size, with significant differences in both the β-TCP/PLLA/PGA–plain media group (p = 0.002) and β-TCP/PLLA/PGA–1 × REC-CM group (p = 0.02), indicating a strong early osteogenic response. By week 4, all groups showed increased defect reduction; however, the β-TCP/PLLA/PGA–10 × REC-CM group maintained superior performance with the most significant defect closure compared with the β-TCP/PLLA/PGA–plain media group (p = 0.003) and β-TCP/PLLA/PGA–1 × REC-CM group (p = 0.03) highlighting the enhanced regenerative effects of REC-CM, particularly at higher concentrations (Fig 4D).

### H&E staining

**Fig 5:**
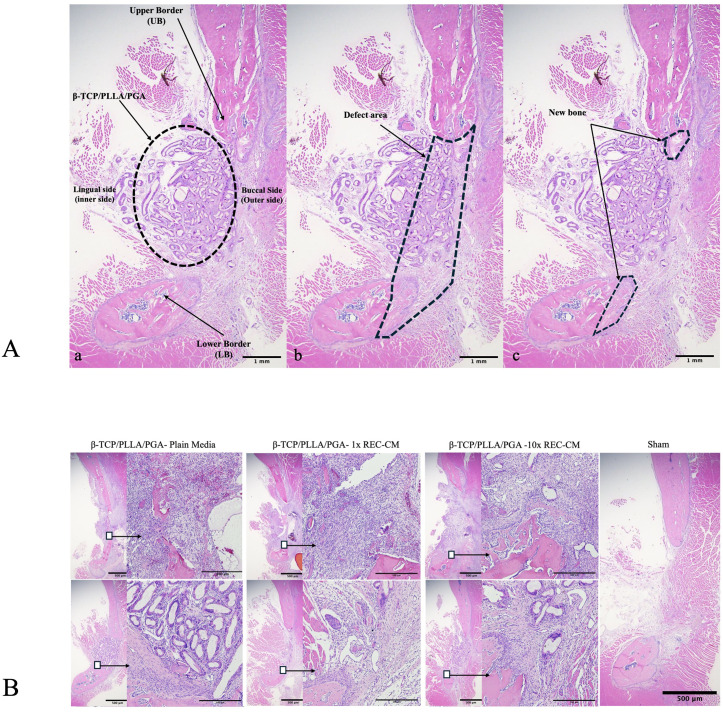
H&E evaluation, and H&E view of the β-TCP/PLLA/PGA in each group. **(A)** H&E evaluation: (a) Image 1x25 magnification including Upper Border (UB); Lower Border (LB) of the defect and β-TCP/PLLA/PGA nanobiomaterial. (b) Illustration of the defect area. (c) Illustration of New Bone on Upper and Lower border. (B) H&E view of the β-TCP/PLLA/PGA–plain media, β-TCP/PLLA/PGA–1 × REC-CM, and β-TCP/PLLA/PGA–10 × REC-CM groups at weeks 1 and 4, and Sham. Images in each subgroup were taken at ×1.25 and ×20 magnification (from left to right). Image of the sham control group were taken at ×1.25 magnification. (×1.25 magnification, scale bar 500µm × 20 magnification, scale bar = 100µm).

The amount of mature and newly regenerated bone at each time point was determined on stained H&E slides. At week 1, the β-TCP/PLLA/PGA–10 × REC-CM group exhibited the most advanced osteogenesis, with visible mineralized bone tissue, organized cellular structures, and vascularization. The β-TCP/PLLA/PGA–1 × REC-CM group showed moderate early bone formation, whereas the β-TCP/PLLA/PGA–plain media group demonstrated minimal activity dominated by inflammatory and fibrotic tissue. By week 4, the β-TCP/PLLA/PGA–10 × REC-CM group demonstrated extensive bone regeneration with mature bone and the presence of abundant stem cells and osteoblasts at the bone surface, indicating good bioactivity. The β-TCP/PLLA/PGA–1 × REC-CM group showed significant improvement with active osteoid deposition, whereas the β-TCP/PLLA/PGA–plain media group exhibited slow and incomplete bone regeneration with sparse newly formed bone and limited cellular activity [27,28]. These findings emphasize the superior efficacy of β-TCP/PLLA/PGA–10 × REC-CM in promoting rapid and robust bone regeneration (Fig 5).

### IHC analyses: expression of different biomarkers

#### Runx2 expression.

The β-TCP/PLLA/PGA–10 × REC-CM group consistently exhibited the highest levels of Runx2 expression. At week 1, the β-TCP/PLLA/PGA–10 × REC-CM group showed significantly greater Runx2 nuclear staining and cell activity than the-TCP/PLLA/PGA–plain media group and β-TCP/PLLA/PGA–10 × REC-CM group, indicating early activation of osteogenesis. By week 4, Runx2 expression remained the highest in the β-TCP/PLLA/PGA–10 × REC-CM group, with intense staining and widespread Runx2-positive cells, reflecting sustained osteogenic progression. The β-TCP/PLLA/PGA–plain media group demonstrated minimal Runx2 expression at both time points, whereas the β-TCP/PLLA/PGA–1 × REC-CM group displayed a moderate increase over time.

**Fig 6:**
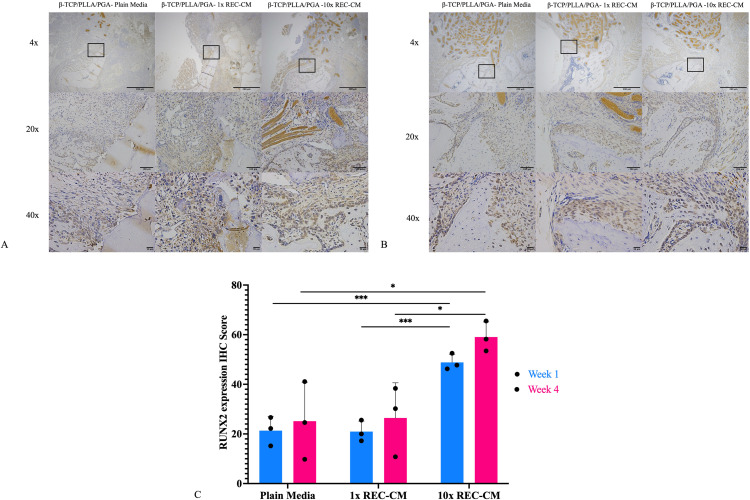
Runx2 immunohistochemistry and graph. **(A)** Runx2 expression in the β-TCP/PLLA/PGA–plain media, β-TCP/PLLA/PGA–1 × REC-CM, and β-TCP/PLLA/PGA–10 × REC-CM groups at week 1 (×4 magnification, scale bar = 200 µm; × 20 magnification, scale bar = 100 µm; × 40 magnification, scale bar = 50 µm). **(B)** Runx2 expression in the β-TCP/PLLA/PGA–plain media, β-TCP/PLLA/PGA–1 × REC-CM, and β-TCP/PLLA/PGA–10 × REC-CM groups at week 4 (×4 magnification, scale bar = 200 µm; × 20 magnification, scale bar = 100 µm; × 40 magnification, scale bar = 50 µm). **(C)** At both time points, the β-TCP/PLLA/PGA–10 × REC-CM group showed significantly higher expression (***p < 0.0002; *p < 0.05).

With respect to IHC-OD scoring at week 1, the β-TCP/PLLA/PGA–10 × REC-CM group had significantly higher scores than the β-TCP/PLLA/PGA–plain media group (p = 0.0001) and β-TCP/PLLA/PGA–1 × REC-CM group (p = 0.0001), suggesting early activation of Runx2-mediated osteogenesis. By week 4, the scores of all groups increased; however, the β-TCP/PLLA/PGA–10 × REC-CM group maintained a statistically significant lead over the β-TCP/PLLA/PGA–plain media group (p = 0.04) and β-TCP/PLLA/PGA–1 × REC-CM group (p = 0.045) reflecting its sustained and enhanced Runx2 expression. These results emphasized the efficacy of β-TCP/PLLA/PGA–10 × REC-CM in activating Runx2 and promoting osteogenesis (Fig 6A–C).

#### LepR expression.

IHC staining for leptin receptor (LepR), a marker associated with osteogenic progenitor activity, was observed at weeks 1 and 4. In the β-TCP/PLLA/PGA–plain media group, LepR expression was minimal, with sparse staining, indicating low progenitor activation. The β-TCP/PLLA/PGA–1 × REC-CM group showed moderate LepR expression with localized staining in specific regions, reflecting increased osteogenic progenitor activity compared to the control. The β-TCP/PLLA/PGA–10 × REC-CM group exhibited intense and widespread LepR staining, particularly at week 4, highlighting robust progenitor activation and recruitment around the scaffold. This progression was most evident in the β-TCP/PLLA/PGA–10 × REC-CM group, which showed a substantial increase in staining density and distribution over time.

**Fig 7:**
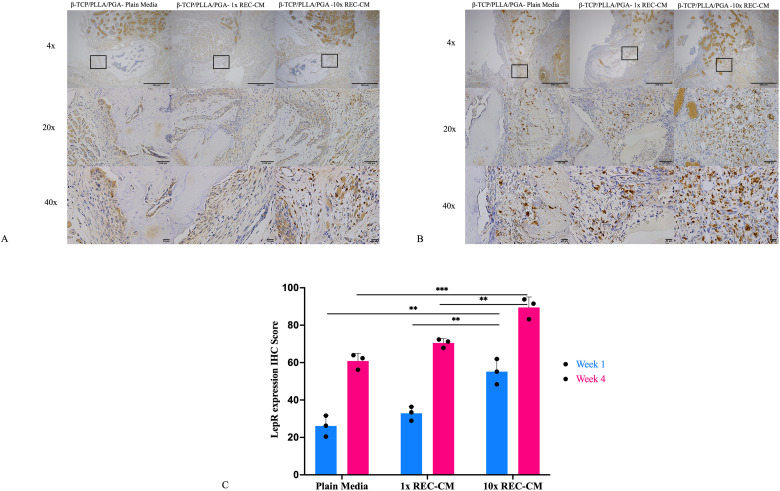
LepR immunohistochemistry and graph. **(A)** LepR expression in the β-TCP/PLLA/PGA–plain media, β-TCP/PLLA/PGA–1 × REC-CM, and β-TCP/PLLA/PGA–10 × REC-CM groups at week 1 (×4 magnification, scale bar = 200 µm; × 20 magnification, scale bar = 100 µm; × 40 magnification, scale bar = 50 µm). **(B)** LepR expression in the β-TCP/PLLA/PGA–plain media, β-TCP/PLLA/PGA–1 × REC-CM, and β-TCP/PLLA/PGA–10 × REC-CM groups at week 4 (×4 magnification, scale bar = 200 µm; × 20 magnification, scale bar = 100 µm; × 40 magnification, scale bar = 50 µm). **(C)** At both time points, the β-TCP/PLLA/PGA–10 × REC-CM group showed significant differences from the other groups (**p < 0.0002; *p < 0.05).

At week 1, the IHC-OD score of the β-TCP/PLLA/PGA–10 × REC-CM group was significantly higher than those of the β-TCP/PLLA/PGA–plain media group (p = 0.002) and β-TCP/PLLA/PGA–1 × REC-CM group (p = 0.007), indicating early activation of LepR-positive osteoprogenitors. At week 4, all groups exhibited increased LepR expression; however, the β-TCP/PLLA/PGA–10 × REC-CM group maintained higher scores than the β-TCP/PLLA/PGA–1 × REC-CM group (p = 0.005) and β-TCP/PLLA/PGA - Plain CM (p = 0.0005) reflecting the sustained activation of osteogenic progenitors (Fig 7 A–C).

#### OCN expression.

At week 1, IHC staining for OCN, a late marker of bone formation, revealed that the β-TCP/PLLA/PGA–plain media group exhibited minimal OCN expression, with weak staining primarily localized in isolated regions, indicating limited bone matrix maturation. The β-TCP/PLLA/PGA–1 × REC-CM group showed moderate OCN staining, suggesting enhanced matrix deposition and early bone formation. The β-TCP/PLLA/PGA–10 × REC-CM group demonstrated the strongest OCN expression at week 1, with widespread and intense staining, indicating good bone formation. By week 4, OCN expression increased in all groups. The β-TCP/PLLA/PGA–plain media group showed limited progression with sparse and faint staining, whereas the β-TCP/PLLA/PGA–1 × REC-CM group displayed moderate staining with enhanced bone matrix maturation. The β-TCP/PLLA/PGA–10 × REC-CM group maintained the highest OCN expression, with dense and extensive staining, reflecting advanced and mature bone matrix formation.

**Fig 8:**
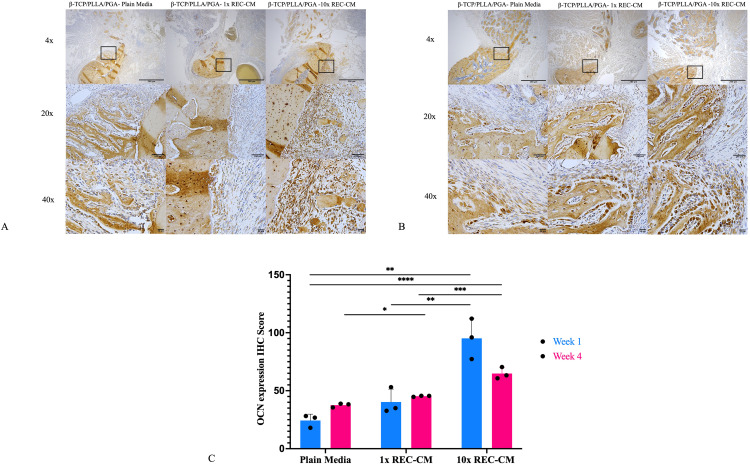
Osteocalcin immunohistochemistry and graph. **(A)** OCN expression in the β-TCP/PLLA/PGA–plain media, β-TCP/PLLA/PGA–1 × REC-CM, and β-TCP/PLLA/PGA–10 × REC-CM groups at week 1 (×4 magnification, scale bar = 200 µm; × 20 magnification, scale bar = 100 µm; × 40 magnification, scale bar = 50 µm). **(B)** OCN expression in the β-TCP/PLLA/PGA–plain media, β-TCP/PLLA/PGA–1 × REC-CM, and β-TCP/PLLA/PGA–10 × REC-CM groups at week 4 (×4 magnification, scale bar = 200 µm; × 20 magnification, scale bar = 100 µm; × 40 magnification, scale bar = 50 µm). **(C)** At both time points, the IHC-OD score of the β-TCP/PLLA/PGA–10 × REC-CM group for OCN showed significant differences from the other groups (***p < 0.0002; **p < 0.0002; *p < 0.05).

Quantitative IHC-OD scoring, aligned with the staining patterns, revealed increased OCN expression. At week 1, the β-TCP/PLLA/PGA–10 × REC-CM group had significantly higher scores than the β-TCP/PLLA/PGA–plain media group (p = 0.001) and β-TCP/PLLA/PGA–1 × REC-CM group (p = 0.005), implying accelerated bone matrix deposition. At week 4, all groups showed increased scores; however, the β-TCP/PLLA/PGA–10 × REC-CM group remained significantly superior, exhibiting the highest OCN expression compared with the-TCP/PLLA/PGA–1 × REC-CM group (p = 0.0001) and β-TCP/PLLA/PGA–plain media group (p = 0.0007) (Fig 8A–C).

#### Periostin expression.

At week 1, IHC staining for periostin, a key marker involved in bone remodeling and regeneration, revealed that the β-TCP/PLLA/PGA–plain media group exhibited weak periostin expression, with limited staining localized to the periphery of the scaffold, indicating minimal remodeling activity. The β-TCP/PLLA/PGA–1 × REC-CM group showed moderate periostin expression, with more prominent staining distributed near the scaffold interface, reflecting enhanced remodeling. In contrast, the β-TCP/PLLA/PGA–10 × REC-CM group displayed the highest periostin expression, with widespread and intense staining, signifying robust bone remodeling and matrix interactions. At week 4, the β-TCP/PLLA/PGA–plain media group maintained weak periostin expression with minimal progress. In contrast, the β-TCP/PLLA/PGA–1 × REC-CM group showed enhanced periostin localization, indicating sustained remodeling activity.

**Fig 9:**
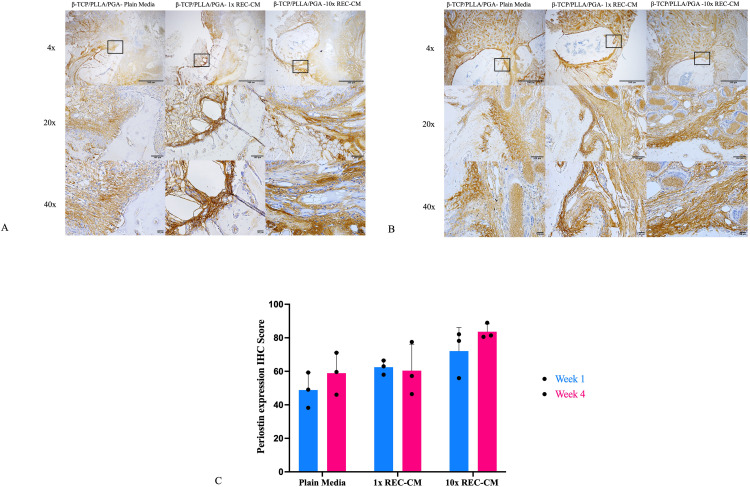
Periostin immunohistochemistry and graph. **(A**) Periostin expression in the β-TCP/PLLA/PGA–plain media, β-TCP/PLLA/PGA–1 × REC-CM, and β-TCP/PLLA/PGA–10 × REC-CM groups at week 1 (×4 magnification, scale bar = 200 µm; × 20 magnification, scale bar = 100 µm; × 40 magnification, scale bar = 50 µm). **(B)** Periostin expression in the β-TCP/PLLA/PGA–plain media, β-TCP/PLLA/PGA–1 × REC-CM, and β-TCP/PLLA/PGA–10 × REC-CM groups at week 4 (×4 magnification, scale bar = 200 µm; × 20 magnification, scale bar = 100 µm; × 40 magnification, scale bar = 50 µm). **(C)** At both time points, the IHC-OD score of the β-TCP/PLLA/PGA–10 × REC-CM group for periostin showed a difference, but only at week 4.

Quantitative IHC-OD scoring at week 1 indicated that the β-TCP/PLLA/PGA–10 × REC-CM group had the highest periostin expression scores, significantly exceeding those of the β-TCP/PLLA/PGA–plain media group and β-TCP/PLLA/PGA–1 × REC-CM group. At week 4, all groups exhibited a slight increase in periostin expression. Nonetheless, the β-TCP/PLLA/PGA–10 × REC-CM group maintained the highest scores compared to the β-TCP/PLLA/PGA–1 × REC-CM group and β-TCP/PLLA/PGA–plain media group, though it was statistically insignificant (Fig 9A–C).

## Discussion

Restoring critical defects in the maxillofacial region poses significant challenges owing to the intricate and unique anatomical features, irregular bone structures, and the presence of a masticatory system with exposure to constant mechanical pressure [29,30]. These factors require regenerative strategies that ensure structural integration of the synthetic biomaterial and address the biomechanical demands unique to this region [31]. The “Diamond Concept” framework introduced by Giannoudis et al., offers a holistic approach to enhancing the healing of bony defects by integrating five essential elements: osteoconductive scaffolds, mechanical stability, osteogenic cells, osteoinductive growth factors, and vascularization [32]. Osteoconductive scaffolds provide a structural framework for cell attachment and growth, and their mechanical stability ensures an optimal environment for healing. Osteogenic cells form the foundation for bone formation, and osteoinductive growth factors activate these cells to promote regeneration. In addition, as highlighted by Osypko et al., vascularization plays a crucial role in supporting osteogenesis by delivering nutrients and recruiting osteoconductive and osteogenic cells. Insufficient vascularization can hinder healing, whereas mechanical stability, along with a conducive host microenvironment, ensures continuous regeneration [33].

### Osteoinductive and osteoconductive capability of combined β-TCP/PLLA/PGA and REC-CM

Osteogenic cells are fundamental to bone regeneration, serving as progenitors that differentiate into osteoblasts, and are responsible for bone matrix synthesis and mineralization. Trompet et al. emphasized the importance of homogeneous and scalable osteogenic cell populations to ensure consistency and effectiveness of bone repair [34]. Similarly, Song et al. demonstrated that engineered scaffolds enhanced osteogenic differentiation and proliferation [35]. Despite these advances, traditional MSCs face persistent challenges such as heterogeneity, senescence, and donor variability, limiting their clinical applicability [36,37]. The introduction of RECs, commercialized as PuREC®, addresses these limitations by offering a highly purified and homogeneous source of MSCs. Compared with conventional MSCs, which are often hampered by donor variability and senescence, REC demonstrate superior consistency in differentiation capabilities, higher resistance to senescence, and retain their regenerative properties even after extensive *in vitro* expansion. Thus, REC is a reliable and scalable option for therapeutic applications in bone regeneration. In this study, the osteogenic potential of the 10 × REC-CM groups amplified Runx2, Osteocalcin, and Periostin marker expression. These results align with the findings of Komori et al., who reported the critical role of Runx2 in osteoblast differentiation and bone matrix production. Elevated VEGF and TGF-β1 levels also facilitated vascularization and matrix deposition, further supporting the regenerative process [38].

Osteoconductive scaffolds play a vital role in bone regeneration by providing a structural framework that facilitates cell attachment, proliferation, and differentiation. These scaffolds act as temporary matrices that guide new bone formation while being integrated into the host tissue. A critical feature of effective scaffolds is their ability to mimic the microarchitecture of natural bone, incorporating porosity and interconnectivity to support vascularization, nutrient diffusion, and bone bridging [39]. β -TCP/PLLA/PGA in a previous study displayed seamless ability to integrate with host bone tissue [12]. The findings on β-TCP/PLLA/PGA in bone regeneration in a rabbit spinal fusion model align with the observations from our study [40]. Mechanical stability is an equally crucial factor beyond these essential features, particularly when addressing the complex biomechanical demands of critically sized bone defects. Restoration and reconstruction of maxillofacial bone defects are challenging because the donor material needs to endure high masticatory pressure. This stability is essential to minimize micromotion at the defect site, thereby enabling uninterrupted cellular activity and matrix deposition. This study identifies the mechanical stability of the β-TCP/PLLA/PGA nanobiomaterial as a limitation. Although the material displayed less spillover from the defect region in this experiment, the biomechanical properties of the scaffold may require further optimization to withstand dynamic and high-pressure environments. Currently, it is FDA-approved and is more suitable for application in walled defects of maxillofacial applications in humans.

**Fig 10:**
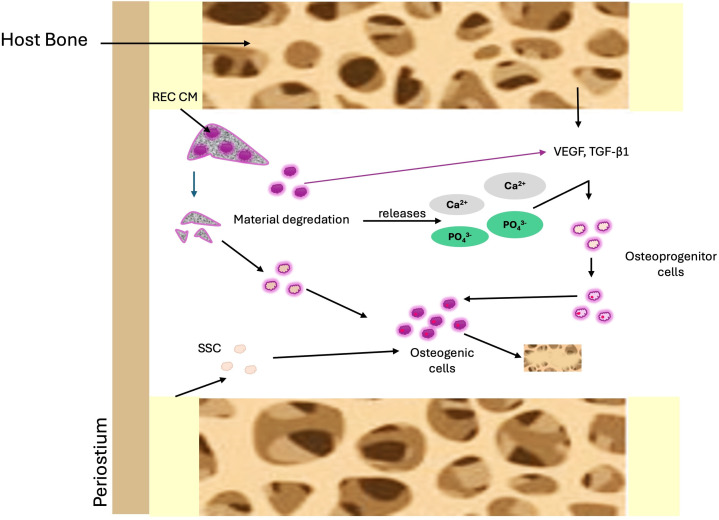
Mechanism of bone regeneration induced by calcium phosphate ion and REC-CM. For a more detailed explanation, refer to text.

The β-TCP component provides an osteoconductive scaffold that gradually releases Ca^2+^ and PO_4_^3-^, which attract progenitor cells from the host bone marrow and support mineralization. The PLLA/PGA components offer initial mechanical stability and degrade over time, creating space for new bone formation. The incorporation of REC-CM, which is rich in VEGF and TGF-β1, further amplifies this process by accelerating vascularization and osteogenic differentiation. VEGF promotes the formation of new blood vessels, ensuring a steady supply of oxygen and nutrients, whereas TGF-β1 enhances the recruitment and differentiation of MSCs into osteoblasts. This combination results in faster bone tissue formation, improved integration with the surrounding bone, and more efficient transition from the scaffold to natural bone. Ultimately, the scaffold gradually degrades while being replaced by fully functional vascularized bone, leading to improved healing outcomes. Fig 10 presents our hypothesis regarding the probable mechanism of bone regeneration in our experiment.

### Role of REC-CM in osseous tissue regeneration

Osugi et al. used MSC-CM in a rat calvarial bone defect, which resulted in a high induction of bone regeneration with upregulated OCN and Runx2 [41]. Takeuchi et al. suggested that exosomes containing MSC-CM enhanced osteogenesis and angiogenesis with high bone regeneration [42]. Ogata et al. demonstrated that MSC-CM accelerated stem cell migration and triggered early bone regeneration by monitoring rat MSC migration toward the local location where MSC-CM was implanted [43]. The concentration of the CM is important for cell proliferation. Studies on MSC-derived CM observed optimal effects at concentrations like 250 µg/mL, beyond which no significant gains in therapeutic efficacy were noted by both Novella et al. and Yao et al. [44,45]. These findings align with the principle that higher concentrations of CM enhance osteogenic and regenerative outcomes, as observed in similar studies using different models and applications. Park et al. investigated lyophilized CM at varying concentrations (1, 5, and 10 µg/mL) and discovered a dose-dependent impact [46]. Paschalidis et al. also evaluated two concentrations of fresh CM for DPSC proliferation (namely, 50% and 100%) and observed that 50% was more substantial [47]. A previous study on extracellular vesicles in equine MSCs revealed that extracellular vesicular productivity plateaued after a 7-day expansion period in 3D bioreactor cultures, indicating a threshold for maximizing production and therapeutic efficacy [48].

The plateau observed in this study with 10 × REC-CM further emphasizes the importance of concentration optimization. The 10 × REC-CM group in our study demonstrated superior bone regenerative potential, as evidenced by increased BMD and BV/TV ratio and the upregulation of key osteogenic markers, including Runx2, osteocalcin, and leptin. Periostin is a vital component of the periosteum and is essential for bone regeneration. In addition to promoting MSC migration and proliferation, it also plays a role in activating skeletal stem cells and depositing cartilage and bone [49–51]. The alignment of periosteal fibers, which is essential for the structural integrity and functionality of tissues, is linked to periostin expression. This alignment facilitates the recruitment and differentiation of cells required for bone repair [52]. According to Yuan et al., periostin regulates bone metabolism and affects BMD [53]. Periostin deposition follows the fiber architecture of the β-TCP/PLLA/PGA scaffold. These findings are consistent with the elevated periosteum observed in our study.

We observed that the 10 × concentration of REC-CM used in this study was optimal because it contained sufficient growth factors to support regeneration. While increasing the CM concentration generally amplifies osteogenic responses, a saturation point may occur where additional concentration increments yield diminishing returns. This phenomenon is likely influenced by cellular receptor saturation and bioavailability of growth factors and cytokines within the CM. In our study, we injected REC-CM only once during scaffold implantation; multiple injections at weekly intervals would be more suitable for long-term evaluation. The 10 × REC-CM resulted in elevated levels of VEGF and TGF-β1. The β-TCP/PLLA/PGA-REC-CM composite exemplifies synergistic action as evidenced by the enhanced expression of osteogenic markers critical for sustained regeneration. Additionally, the ability of the scaffold to support both the early and sustained phases of bone healing mirrors the findings from the ReBOSSIS studies, where rapid host tissue integration was a key advantage [40]. Overall, scaffolds that combine mechanical stability with bioactivity enhancement can address the dual challenges of structural integrity and biological functionality in bone regeneration.

### Importance of vascularization

Vascularization plays a pivotal role in bone regeneration as it ensures the delivery of oxygen, nutrients, and essential cellular components to the site of bone healing. The integration of vascularization with osteogenic strategies is critical for successful bone repair, particularly in scaffolds designed for maxillofacial reconstruction. Recent advances in tissue engineering have highlighted the necessity of coupling osteogenesis and vasculogenesis to achieve functional bone repair. VEGF-mediated angiogenesis facilitates the formation of vascular networks that support the recruitment of osteoprogenitor cells, a process essential for sustained bone regeneration [54–56]. Ha et al., provided insights into scaffold designs that mimic natural bone’s vascularized architecture, a concept reflected in the interconnected porous structure of our β-TCP/PLLA/PGA nanobiomaterial [39]. Gerhardt et al. reported similar vascularization effects in mesoporous bioactive scaffolds loaded with pro-angiogenic factors [57]. The above literature corroborates our findings regarding the significantly high VEGF and TGF-β1 results in promoting angiogenesis and osteogenesis within the β-TCP/PLLA/PGA-REC-CM framework.

β-TCP/PLLA/PGA scaffold combined with REC-CM at 10 × concentration represents an innovative approach that integrates structural and regenerative benefits. The cotton-like structure of this composite not only ensures adaptability to irregular defects but also provides the mechanical support necessary to maintain scaffold integrity during the early phases of healing. Incorporating bioactive components like REC-CM further amplifies its regenerative potential, delivering growth factors such as TGF-β1 and VEGF to enhance osteogenesis and angiogenesis.

### Limitations

Because this was a pilot study, we employed only a limited number of rats in each group. The results of statistical testing can be substantially improved by analyzing more specimens. In our study, REC-CM was injected only once during surgery. In our follow-up study, we aimed to provide repeated injections and analyze the outcomes. We had a shorter time-point analysis in this study, namely, 1 week and 4 weeks; increasing the time-point would allow for a better evaluation of results in the long term.

### Future perspectives

In our study, CM containing bovine serum was used to facilitate the survival and proliferation of REC *in vitro*. In future studies, we intend to use serum-free media supplemented with additional growth factors to overcome this limitation. The lack of immunogenicity associated with REC-CM has interesting clinical applications, given that human stem cell-derived CM can be lyophilized and applied to wound healing and tissue regeneration. We aim to further investigate the molecular properties and regenerative mechanisms of REC-CM for therapeutic translation.

We would also like to state that we conducted this study as preliminary research in order to observe the effects of concentrated CM alongside the β-TCP/PLLA/PGA nanobiomaterial. It is noteworthy that the 10x REC-CM was added to the site of the critical defect at only one-time point, i.e., during surgery. Based on our results, it can be said that conditioned media therapy potentially has huge benefits in therapeutic applications. The conditioned media can be concentrated and stored as frozen stock, which can then be repeatedly administered in maxillofacial bones, especially at set time points as required. However, we do concur that future research incorporating the disadvantages of the current study will provide further clarification and shed light on the long-term effects of conditioned media application in bone regeneration.

The time period of follow-up assessment in our study was shorter (1 and 4 weeks) as this is a preliminary study wherein, we wanted to ascertain early bone regeneration response caused by one-time application of Conditioned Media. We will conduct further follow-up studies in order to determine the resorption rate of the nanobiomaterial and the results elucidated upon changing the method, volume variation, time, and the number of Conditioned Media applications to obtain effective solution to induce faster and more upregulated bone regeneration.

## Conclusion

Our study highlights the regenerative potential of β-TCP/PLLA/PGA nanobiomaterial scaffolds combined with REC-CM in enhancing bone regeneration. Our findings demonstrate that β-TCP/PLLA/PGA–10 × REC-CM consistently outperformed other groups, exhibiting superior bone volume and mineral density, as well as upregulated expression of key osteogenic markers such as Runx2, Osteocalcin, LepR, and Periostin. These outcomes reflect the robust osteogenic and angiogenic capabilities of the β-TCP/PLLA/PGA–10 × REC-CM combination, establishing its effectiveness in promoting rapid and sustained bone regeneration.

The study elaborates on the synergistic role of the β-TCP/PLLA/PGA scaffold in providing a supportive framework for new bone formation. Simultaneously, REC-CM delivered concentrated bioactive factors that drive osteogenesis and vascularization. This integrated approach addresses the limitations of traditional treatments, such as donor site morbidity and limited regeneration, and offers a scalable and immune-compatible solution for bone repair.

These findings lay the groundwork for innovative therapeutic strategies in reconstructive surgery, with the β-TCP/PLLA/PGA–10 × REC-CM combination showing significant promise for clinical translation. Future research should validate these results using larger models and optimize scaffold designs to maximize regenerative outcomes, thereby advancing the fields of tissue engineering and regenerative medicine.

## Supporting information

S1 Fig Evaluation of BV/TV ratio. The 4-mm critical size defect and binary images of β-TCP/PLLA/PGA-REC-CM for evaluation of BV/TV using ImageJ software.
(TIF)

S2 Fig Evaluation of IHC-OD. IHC-OD evaluation was performed using ImageJ software. Log display showing the score for an example image.
(TIF)

S1 Table Full research dataset. The complete dataset used for statistical analysis and figure preparation.
(XLSX)

S2 Table Shapiro-Wilk’s normality test results. Results of Shapiro–Wilk’s test performed on each dataset to assess normality. A p-value > 0.05 indicates the data did not significantly deviate from a normal distribution.
(XLSX)
