# Supplementary figures and images for: Evaluation of bone regenerative and bioactive potential of β-TCP/PLLA/PGA nanobiomaterial in conjunction with concentrated conditioned media from rapidly expanding clone (REC) of mesenchymal stem cells: A pilot animal study
Source: PLoS One. 2025 Oct 9;20(10):e0332531. doi: 10.1371/journal.pone.0332531 (PMC12510494; doi:10.1371/journal.pone.0332531)

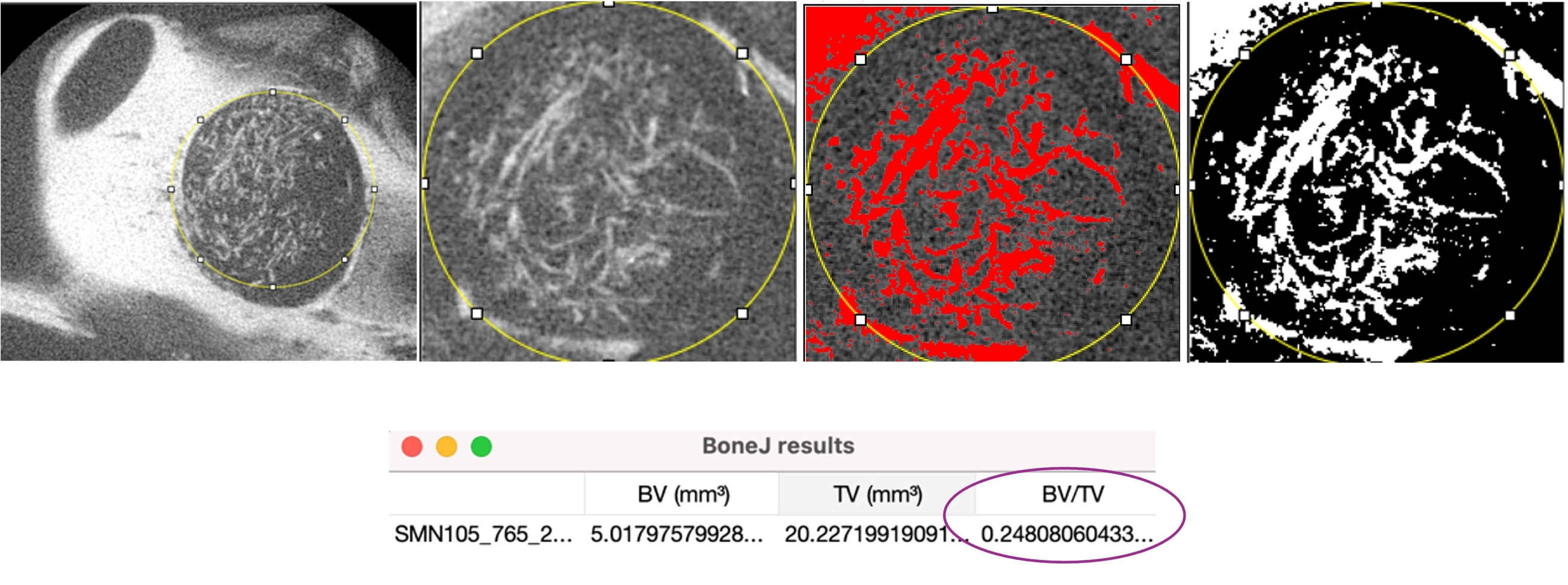

Supplement: S1 Fig — The 4-mm critical size defect and binary images of β-TCP/PLLA/PGA-REC-CM for evaluation of BV/TV using ImageJ software. (TIF) [file pone.0332531.s001.tif]

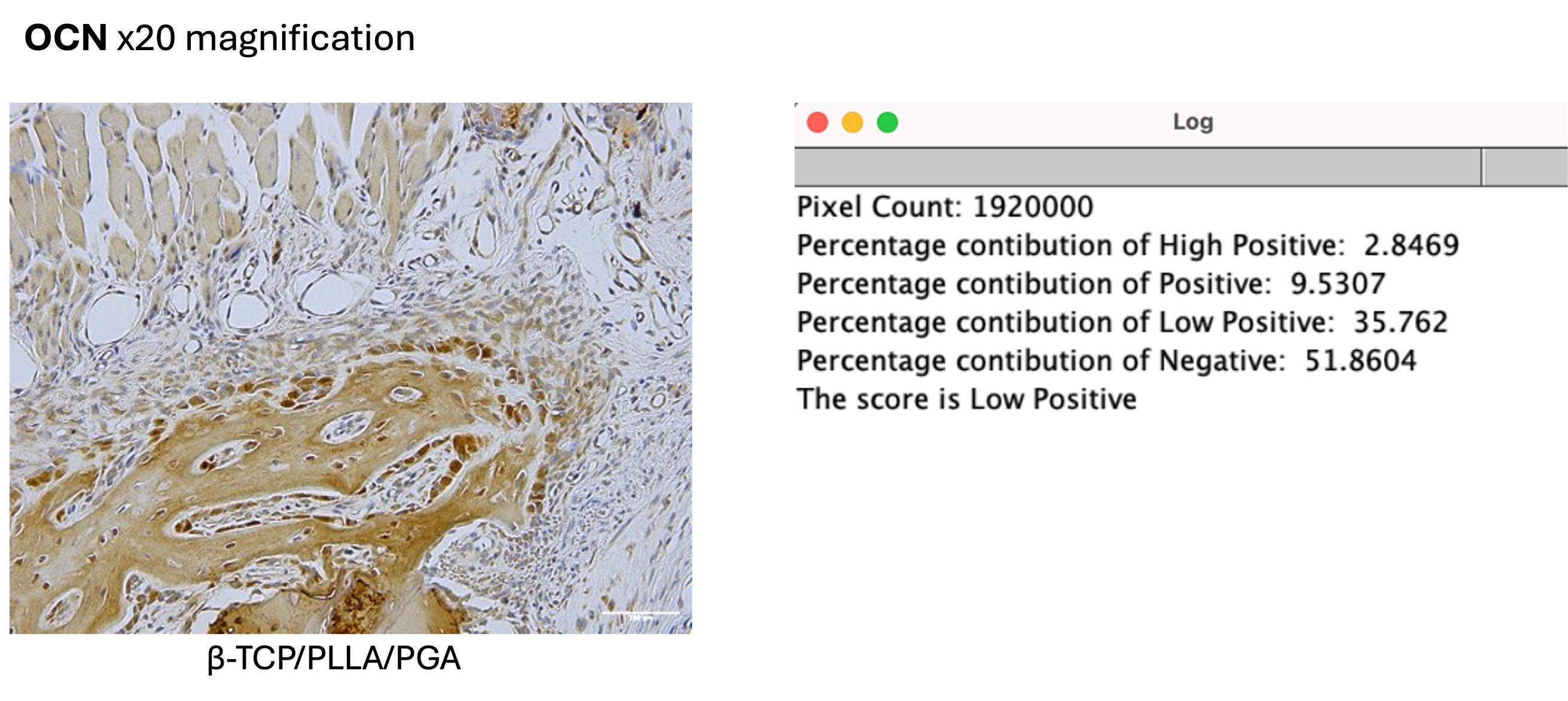

Supplement: S2 Fig — IHC-OD evaluation was performed using ImageJ software. Log display showing the score for an example image. (TIF) [file pone.0332531.s002.tif]
